# The AI-augmented researcher: Optimizing systematic reviews in ergonomics

**DOI:** 10.1177/10519815261478123

**Published:** 2026-10-16

**Authors:** David Ernesto Puentes-Lagos, Karen Lange-Morales, Aida Josefina Rojas-Fajardo

**Affiliations:** 1 Escuela de Diseño Industrial, 28021 Universidad Nacional de Colombia, Bogotá, Colombia; 2 Doctorado en Ergonomía, 28006 Universidad del Valle, Cali, Colombia

**Keywords:** artificial intelligence, ergonomics, human factors, work, technology, information storage and retrieval, systematic review, efficiency

## Abstract

**Background:**

The emergence of Academic Artificial Intelligence (AAI) agents is reshaping Systematic Literature Reviews (SLR) by expanding evidence screening and synthesis beyond Boolean, metadata-based searches, but its use requires a clear, PRISMA-aligned human oversight model to avoid compromising research integrity.

**Objective:**

This opinion article proposes the “AI-augmented researcher” as a multidimensional framework that synergizes human analytical rigor with Large Language Models (LLMs). Using a physical ergonomics case study, the paper establishes a normative integration strategy that demonstrates how AAI can accelerate evidence synthesis while maintaining strict PRISMA compliance under explicit human oversight.

**Methodology:**

A Workflow Comparative Analysis (WCA) applying a parallel-group design to physical ergonomics SLR. The procedure synchronized two approaches: 1) A “classical” SLR workflow, using Boolean syntax and paratextual screening (Metadata) across indexed databases; and 2) An AAI-driven workflow, utilizing LLM-based immersion via natural language prompting. Comparative metrics analyzed included execution time, record processing volume, and the identification of emerging trends—such as Ergonomics 4.0—benchmarked against the PRISMA 2020 protocol.

**Results:**

The AAI-augmented workflow reduced execution time by 96% (39 h to 1.5 h) while increasing record processing by 370%. Qualitatively, iterative prompting fostered a “hermeneutic dialogue,” enabling the detection of complex “Ergonomics 4.0” trends obscured in syntactic searches.

**Conclusions:**

AAI serves as a cognitive enhancer, reconfiguring the researcher from a “data miner” to an “evidence curator”. While efficiency is optimized, strict human oversight remains indispensable to mitigate algorithmic hallucinations and ensure ethical scientific integrity.

## Introduction: Boolean constraint

This propositional paper uses a physical ergonomics case as proof-of-concept to illustrate an AI integration strategy, rather than empirical generalization. The emergence of Academic Artificial Intelligence (AAI) marks a turning point for Systematic Literature Reviews (SLR) and the PRISMA^1^: fundamental evidence pillars in ergonomics.^2,3^ Traditionally SLR has relied on the “Boolean constraint”, syntactic equations that filter only metadata -titles, abstracts and keywords-, acting as a barrier for deep conceptual nuances.^4^ While AAI already offers automation and speed,^5^ it is crucial to question the evolving role of researchers and editorial teams. In this opinion paper, we argue that AAI should be used to accelerate evidence synthesis only within a PRISMA-aligned, human-in-the-loop workflow, where researchers remain accountable for critical appraisal and reporting.

Accordingly, we propose the “AI-augmented researcher” as a pragmatic integration model that combines LLM-enabled semantic screening with explicit human oversight and responsibility. By applying Postman's technological bargain^6^ viewing AI as both a burden and a blessing, we aim to ensure scientific integrity without demonizing technological progress.

## From data miner to evidence curator

Traditional and AAI-driven workflows present distinct methodologies for scientific synthesis^7^ as compared in Table 1. SLRs rely on indexed paratexts – titles, abstracts, and keywords - structured via Boolean operators, often bypass the full document body during the initial screening.^7^ This “thesaurus dependency” creates a bottleneck, requiring a socio-technical mastery and extensive training to ensure reliability among new researchers.

**Table 1.** Comparative workflows for SLR: “traditional” vs AAI.

Feature	Traditional SLR (Boolean-based)	AAI-Augmented SLR (LLM-based)
Search Logic	Syntactic (Keywords & Set Theory)	Semantic (Natural Language & Context)
Data Source	Metadata & Paratexts (Limited)	Full-text Corpus (Deep Immersion)
Main Constraint	“Thesaurus” Bottleneck	Algorithmic Hallucinations
Technical Skill	Mastery of Boolean Operators	Prompt Engineering & Critical Curation
Learning Process	High effort in “Data Mining”	High effort in “Evidence Synthesis”
Global Equity	Limited by Library Subscriptions	Limited by AI Tool Access/Digital Divide

In contrast AAI agents leverage Large Language Models (LLMs) to perform deep immersion into full texts using natural scientific language.^7^ While specialized AAI models offer granular analysis^5,8^ this shift demands new competences in prompting skills and critical validation.^9^ However, a “cognitive debt”^10^ may emerge for doctoral students using these tools as a “black box” shortcut, potentially bypassing the deep mapping of research gaps.^11^ Furthermore, persistent algorithmic hallucinations and the lack of global equity in accessing these sources^12^ create new forms of exclusion that must be addressed.

## The case: physical ergonomics

The Workflow Comparative Analysis (WCA) focused on physical ergonomics methods and tools^13^ suggests a marked difference in workflow speed.

### Efficiency and temporal metrics: a proof-of-concept

In the traditional approach, a team of three doctoral researchers invested 39 h to screen 122 articles based strictly on paratextual metadata (titles, abstracts and keywords).^14^ In contrast, the AAI agent processed an initial pool of 575 records in only 1.5 h, enabling rapid full-text screening. This automated process flagged candidate studies while applying the same eligibility criteria as the human team, reducing total execution time by 96%.^13^ These figures are proof-of-concept, not generalizable performance estimates (Table 2).

**Table 2.** Comparative workflows result of the physical ergonomics case study.

Feature	Traditional SLR (Boolean-based)	AAI-Augmented SLR (LLM-based)
Execution time, records identified, sessions	39 h, 122 papers screening, 4 sessions of work.	1.5 h, 575 papers screening, 1 session of work
Synthesis of results	Thematic classification based on general ergonomics methods (OWAS, RULA, REBA). Focus on musculoskeletal risk prevention through standardized manual observation.	Multi-dimensional synthesis with industrial mapping. Automatic extraction of experimental variables, sample sizes, and specific sectors (Manufacturing, Health, Logistics).
Technical Constraint	Syntactic Bottleneck: Limited by rigid Boolean strings: “Physical ergonomics” AND (Methods OR Technics OR Techniques OR Tools).	Semantic Prompt (NLP): “Act as an ergonomics expert. Conduct a PRISMA 2020 SLR on ‘Physical Ergonomics Methods and technics (2010–2025)’. Analyze full-texts and detect trends.”

### Qualitative synthesis and semantic trend detection

Beyond time efficiency, the AAI-driven workflow enabled a more granular qualitative synthesis in this case. While manual screening often limits results to broad thematic categories, the AAI agent performed a multi-dimensional analysis, classifying findings by industrial sectors (manufacturing, healthcare, logistics) and evaluating evidence quality based on experimental designs. This granular approach identified high-value surface trends that may be missed in metadata-only screening such as “Ergonomics 4.0”^15^ and wearable sensors,^16^ which are typically obscured in traditional metadata-only searches.

Consequently, this augmented workflow redefines the researcher's role from a mechanical “data miner” to a high-level “evidence curator” capable of complex scientific synthesis.^10^ This shift requires existing frameworks, such as PRISMA 2020, to evolve by incorporating new quality protocols that validate AAI-generated outputs, ensuring that technological acceleration does not compromise the development of critical doctoral expertise.^17^

AAI agents and assistants have automatic short-term options: they generate tables, maps, graphs and other academic outputs that speed up the vision and understanding of a field.^6,11^ It can accelerate some research tasks, especially at scale. While manual synchronous analysis of thousands of articles is often utopic or requires months of researcher's labor, in this case, AAI performed this synthesis in minutes, providing a high-resolution overview of the state-of-the-art.

## The hermeneutic dialogue: prompting as a thinking process

Human indispensability is proposed but augmented.^18,19^ As demonstrated in the physical ergonomics case study, LLM-based agents can support semantic synthesis and trend detection, compared to traditional Boolean methods; however, outputs require verification and are sensitive to prompting, tool access, and full-text availability. The “Augmented Researcher” does not replace human judgment; instead, it leverages technology to enhance the depth, speed, and impact of scientific discovery in ergonomics. This shift changes doctoral training from data retrieval to evidence curation.

This shift elevates iterative prompting from a technical command to a profound dialogue, where “semantic immersion” and “agency of meaning” are co-constructed between the researcher and the machine.^20^ The interaction functions as a digital hermeneutic cycle,^21^ where the researcher's pre-understanding of ergonomics is constantly refined through the AAI's ability to navigate full-text corpora. The validity of the evidence rests on this interpretive rigor; the researcher is no longer a passive observer of results, but an active agent in a dialectical exchange that transforms data into structured, critical, and ethical knowledge.^19^

Human oversight is required during initial SLR phases, focusing on: 1) Funding of included studies^22^; 2) Ethics implicit in reviewed documents^19^; 3) Researcher biases within the screened articles.^23^

## Limitations

This study has constraints regarding technological maturity; the reliance on technical documentation from AAI providers stems from the scarcity of peer-reviewed empirical evidence in this emerging field. Furthermore, results are contingent upon 2024 LLM architectures, suggesting that efficiency and error rates may evolve. Finally, the “hermeneutic dialogue” effectiveness depends on the researcher's prompting proficiency and stable high-speed connectivity, highlighting potential gaps in global research equity. LLMs cannot yet reliably appraise bias or study validity. Overreliance may erode doctoral critical-reading skills.

## Conclusions

The “Augmented Researcher” enhances traditional SLRs workflows through AAI-driven synthesis while maintaining PRISMA's indispensable rigor. It is most appropriate when full texts are accessible and outputs are verified. As an emergent technology, AAI requires evolving intermediate controls to mitigate “black box” opacity and oversight of funding and bias. AAI can speed mapping; humans must retain critical appraisal.
